# Comparison the Effect of Ferutinin and 17β-Estradiol on Bone Mineralization of Developing Zebrafish (*Danio rerio*) Larvae

**DOI:** 10.3390/ijms20061507

**Published:** 2019-03-26

**Authors:** Hoda Zare Mirakabad, Mohammad Farsi, Saeed Malekzadeh Shafaroudi, Abdolreza Bagheri, Mehrdad Iranshahi, Nasrin Moshtaghi

**Affiliations:** 1 Department of Biotechnology and Plant Breeding, Ferdowsi University of Mashhad, Mashhad 91775-1163, Iran; ho_za9@mail.um.ac.ir or hodazma313@gmail.com (H.Z.M.); malekzadeh-s@um.ac.ir (S.M.S.); abagheri@um.ac.ir (A.B.); moshtaghi@um.ac.ir (N.M.); 2 Department of Pharmacognosy; Mashhad University of Medical Sciences, Mashhad 91886-17871, Iran; iranshahiM@mums.ac.ir

**Keywords:** ferutinin, 17β-estradiol, zebrafish, LC50, RT-PCR, real-time PCR, histology, chemical screening, skeletal tissue, osteogenesis

## Abstract

There is an urgent need to develop novel drugs for osteoporosis which occurs due to estrogen deficiency. Phytoestrogens derived from medicinal plants would be the best alternative to chemical drugs with harmful side effects. The main purpose of the present study was to investigate the effect of ferutinin compared to 17β-estradiol (E2) on bone mineralization of zebrafish larvae. Regarding the lack of publications, the histology analysis was performed after exposure to E2 to find effective treatment on bone mineralization of developing zebrafish larvae. Then, the larvae were exposed to four concentrations of ferutinin at three time points to assess the mortality, the expression of some related genes and histology of the ceratohyal and hyomandibular of treated larvae. The RT-PCR result of the treatment groups demonstrated the similar expression pattern in the larvae which were exposed to 1.25 μg/mL of ferutinin and 2 µM of E2 at 2 dpf, which confirmed the result of histology analysis. In addition, RT-qPCR of high concentration of ferutinin and E2 demonstrated that *bmp2a/b* and *esr1* were downregulated and upregulated when the larvae were exposed to 5 μg/mL of ferutinin and 10 µM of E2, respectively.

## 1. Introduction

Deficiency of estrogen could change the balance between osteoclast and osteoblast which is the reason for increasing the risk of fracturing bone in menopause women and is defined as a kind of osteoporosis. The Women’s Health Initiative (WHI) showed that Hormone Replace Therapy (HRT) prevents the fracturing bone due to the osteoporosis that is in turn the result of estrogen deficiency, but it could increase the risk of breast cancer as well as cardiovascular diseases in women who take HRT for a long time [1]. ERα is the major ER mediating the effects of estrogen on bone in females and in males; however, ERβ may play a role in estrogen action in the trabecular bone under certain circumstances as a substitute or enhancement of ERα action [2]. The result of [3] demonstrated that among the natural estrogens (estriol, estrone, 17α-estradiol, and 17β-estradiol), 17β-estradiol (E2) was the compound with the highest potency toward the three zebrafish estrogen receptors (zfERα, zfERβ_1_, zfERβ_2_) and among the phytoestrogens (genistein, ferutinin, and liquiritigenin), ferutinin with similar affinity for hERα and hERβ but different activity (full agonist on hERα and partial agonist on hERβ), behaved as full agonist on the three zfERs. Therefore, studies have been focused on phytoestrogens because of their estrogenic activities and lack of adverse side effects associated with estrogens [4].

In recent years, several publications from Italy addressed the concept of ferutinin as an interesting phytoestrogen with an osteoinductive capability with application either for treatment of osteoporosis or in bone tissue engineering [4,5,6,7,8,9,10,11,12,13]. The result also demonstrated the role of ferutinin in the impairment of female sexual function and its effect on promoting proliferation and differentiation in human dental pulp and amniotic fluid stem cells [10,11,12]. The importance of ferutinin has been realized in bone metabolism since it is capable to prevent and treat osteoporosis including estrogen deficiency in ovariectomized rats [5,6,7,8,9]. Ferutinin, through ERα, improves bone reconstruction, when orally administered in rats with a calvaria critical size bone defect, filled with a collagen type 1 and human amniotic fluid stem cells (hAFSCs) construct. This construct leads to an approximately 70% bone reconstruction showing that ferutinin could act as a healing promoting factor, on hAFSCs including osteogenic differentiation [4]. In another study [11], it was tried to explain a possible molecular mechanism for ferutinin-induced osteoblastic differentiation of hAFSCs, through ERα and GPR30, evaluating the role of the MEK/ERK and P13K/Akt signaling pathway, and indicated that ferutinin is able to stimulate both MEK/ERK and P13K/Akt signaling in undifferentiated hAFSCs, although with different timing of the phosphorylation pattern. Moreover, [11] indicated that in the canonical osteoblastic differentiation model, both pathways were involved, but P13K/Akt is required to ferutinin stimulated osteoblastic differentiation through ERα. 

Zebrafish is a well-developed model system for studying both embryonic development and human diseases. The primary advantages of zebrafish for drug discovery include small size, optical transparency, rapid development, large number of their embryos and larvae, as well as their high genetic, physiologic, and pharmacologic similarity to humans, particularly, high similarity with humans in terms of bone architecture, bone cells, matrix proteins, and molecular signaling defined as an ideal in vivo model for the systematic identification of bioactive natural products with therapeutic capability and suitable model for screening of the agents to prevent and treat osteoporosis [14,15,16,17,18,19]. The ovariectomized rat is the most common animal model in the study of anti-osteoporosis medication which was used in the study of [5,6,7,8,9] to find effect of ferutinin on osteoporosis. Zhang et al. [20] used osteoporotic model induced by glucocorticoids as chemical drugs to show anti- osteoporosis effect of Chinese traditional drug after discussing about the disadvantages of using ovariectomised rat. Luo et al. [18] and Vrieze et al. [19] used chemical drugs such as prednisolone and dexamethasone to induce osteoporosis in zebrafish. Luo et al. [18] recommended monitoring bone formation directly in transgenic zebrafish *tg (sp7:egfp)* and inferred to fine and complicated procedure of staining a tiny model for directly observation of bone formation in the model of wild-type zebrafish larvae.

There are several studies which used zebrafish embryos to study the effects of estrogens on organ formation and function [21,22,23] and to trace the effect of E2 on chondrogenesis and expression of related genes [24,25,26]. Pashai Ahi et al. [26] demonstrated E2 mediated differential expression of some genes which involved in craniofacial skeletal development (e.g., *bmp2a/b*) as well as genes co expressed with *esr1*(a ligand-activated receptor for estrogen). However, a significant amount of research has been conducted for the effect of E2 on cartilage development and related gene expression using zebrafish larvae [24,25,26], but there is not any study about the effect of E2 on the ossification of zebrafish.

The drug screening using zebrafish larvae require a system to expose embryos to the compound in culture solution over a specific length of time. The test platform of the screening mainly relies on multi-well plate in which the compounds or culture solution require being renewed and the dead embryos should be removed [27]. While the washing embryos are time-consuming and may injure the embryos, a large and growing body of literature has investigated the flow conditions in microfluidic chip to cultures them [27,28,29,30]. For example, [28] developed a modified 24-well plate with a flow-through system which requires a large volume of the test compound. A research team [29] established a microfluidic chip that docks zebrafish embryos automatically and cultures them under flow conditions, but it does not allow for the timely removal of the dead embryos. Akagi et al. [30] tried to overcome this problem and introduced the 3D multilayer microfluidic system for real-time developmental analysis of zebrafish embryos, but it was impossible to expose the fish eggs to multiple substances at different concentration. Li et al. [27] introduced a microfluidic device to simultaneously evaluate the developmental toxicity of an anti-asthmatic agent on zebrafish embryos and larvae using real-time imaging.

Here, we investigated whether the effect of ferutinin on bone mineralization in vivo is similar to E2 by exposing zebrafish larvae to a range of E2 and ferutinin or not. In this context three sections for the experiments were designed: (i) investigating the effect of E2 on bone mineralization of zebrafish larvae as positive control; (ii) monitoring the developmental toxicity of ferutinin; and (iii) finding the effective concentration of ferutinin on bone mineralization and expression of target genes as main purpose of the study. The positive effect of ferutinin on bone mineralization of wild-type zebrafish would be helpful to increase studies in this area such as investigating the side effect of ferutinin and possibly to introduce it as a drug.

## 2. Results

### 2.1. Effect of E2 on the Phenotype of Bone Mineralization

**Figure 1:**
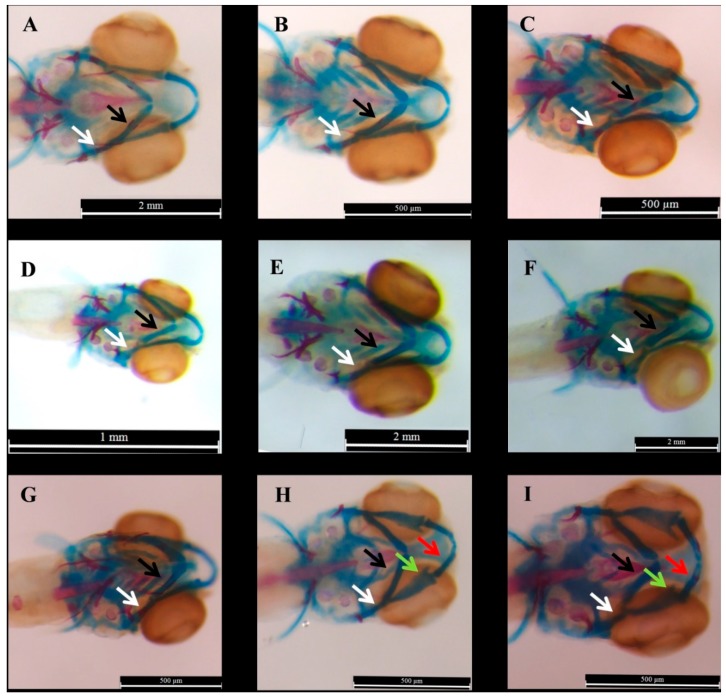
The samples of the morphological changes in the E2-treated zabrafish at 6 dpf which were treated with 2, 10 and 20 µM of E2 compared to DMSO (0.1%) at 1 dpf, 2 dpf, or 3 dpf. Scale bars represent 500 µM, 1 mm, and 2 mm. The blue parts represent ceratohyal and red parts demonstrate mineralized sections. (**A**–**C**) indicate the samples of the larvae which were treated with 2 µM of E2 at 1 dpf, 2 dpf, and 3 dpf, respectively, (**D**–**F**) Show the result of alcian blue-alizarin red staining larvae which were exposed to DMSO (0.1%) at 1 dpf, 2 dpf, and 3 dpf, respectively. (**G**–**I**) represent the larvae which were exposed to E2 at 3 dpf with 10, 20, and 20 µM. The arrows demonstrate some changes: white for hyomandibular, black for ceratohyal, green for palatoquadrate, and red arrows for Meckel.

In the current study, the various morphological changes was observed in the cartilage and bone elements (e.g., ceratohyal, ethmoid plate, palatoquadrate, and Meckel), but we investigated the effect of exposure time and concentration of treatment on the phenotype of ceratohyal and hyomandibular mineralization (Figure 1). 

**Figure 2:**
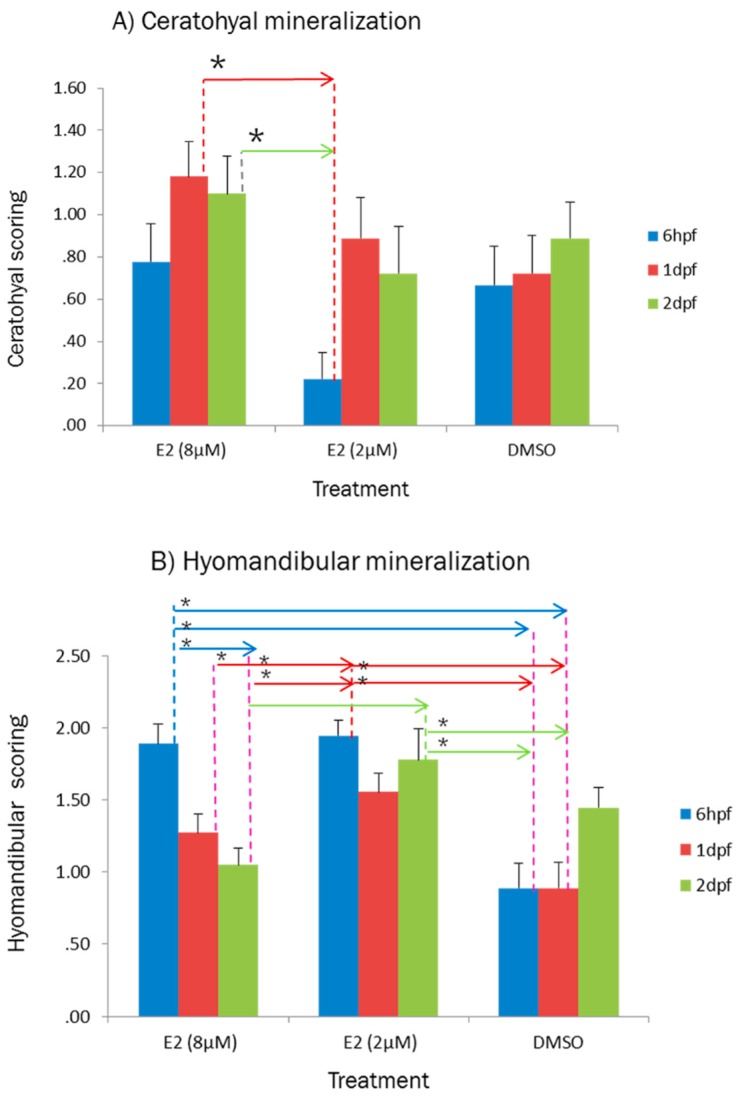
Effect of concentration and exposure time on ceratohyal (**A**) and hyomandibular (**B**) mineralization of 6 dpf zebrafish larvae which were treated with 2 and 8 µM of E2 compared to DMSO (0.1%) at 6 hpf, 1 dpf, and 2 dpf. Bars represent mean +SEM (*n* = 20 fish per group). Samples were analyzed in triplicate. Dashed line and arrow indicate groups with significant difference in mean of bone mineralization which were statistically different (* *p* < 0.05), by multiple comparison of means using one-way ANOVA and Tukey’s post hoc test.

The results of this study showed a trend in bone mineralization of the treated larvae with E2 (Figure 2). The score of ceratohyal mineralization in E2-treated larvae at 1 dpf reached the peak score. Interestingly, increasing concentration of estradiol showed a steady rise in the ceratohyal and steady fall in hyomandibular mineralization. Treating at 1 dpf could decrease mineralization of hyomandibular while it increased ceratohyal mineralization. The ceratohyal mineralization was significantly decreased in the larvae which were treated at 6hpf with 2 µM of E2 compared to which were exposed to 8µM of E2 at 1 dpf and 2 dpf (Figure 2A), but it showed significantly increased in hyomandibular mineralization compared to 8 µM of E2 at 1 dpf and 2 dpf and DMSO at 6 hpf and 1 dpf (Figure 2B).

**Figure 3:**
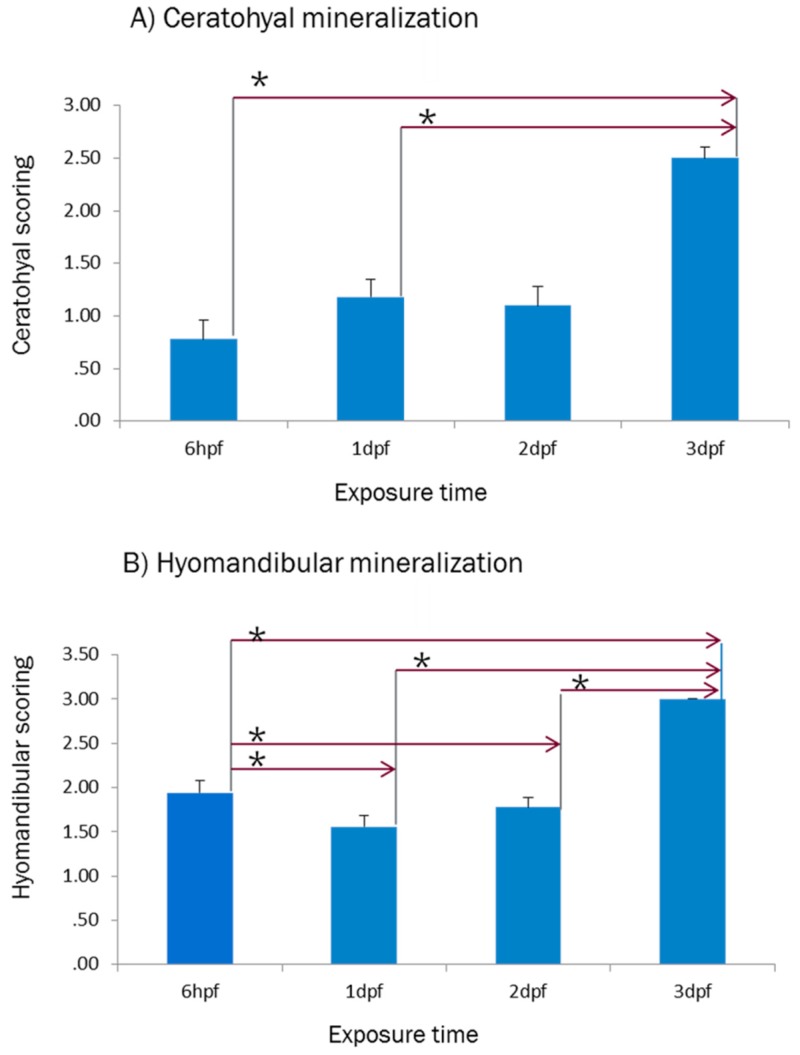
Effect of exposure time on ceratohyal (**A**) and hyomandibular (**B**) mineralization of zebrafish larvae treated with 8 µM of E2 at 6 hpf, 1 dpf, 2 dpf, and 3d pf. Bars represent mean +SEM (*n* = 20 fish per group). Dashed line and arrow indicate groups with significant difference in mean comparison (one-way ANOVA followed by Tukey’s post hoc test, * *p* < 0.05). Samples were analyzed in triplicate. Abbreviations: hpf indicates hours post fertilization, dpf represents days post fertilization.

On the other hand, the results of multiple comparisons showed that there is a significant difference between treating before and after 24 hpf (1 dpf) in bone mineralization of larvae. The bone mineralization of the larvae which were treated with 8 µM of E2 was assessed to clarify effect of exposure time and the result indicated that bone mineralization was increased significantly in 3 dpf-treated larvae (Figure 3). 

### 2.2. Effect of Ferutinin on Mortality of Zebrafish Larvae

**Figure 4:**
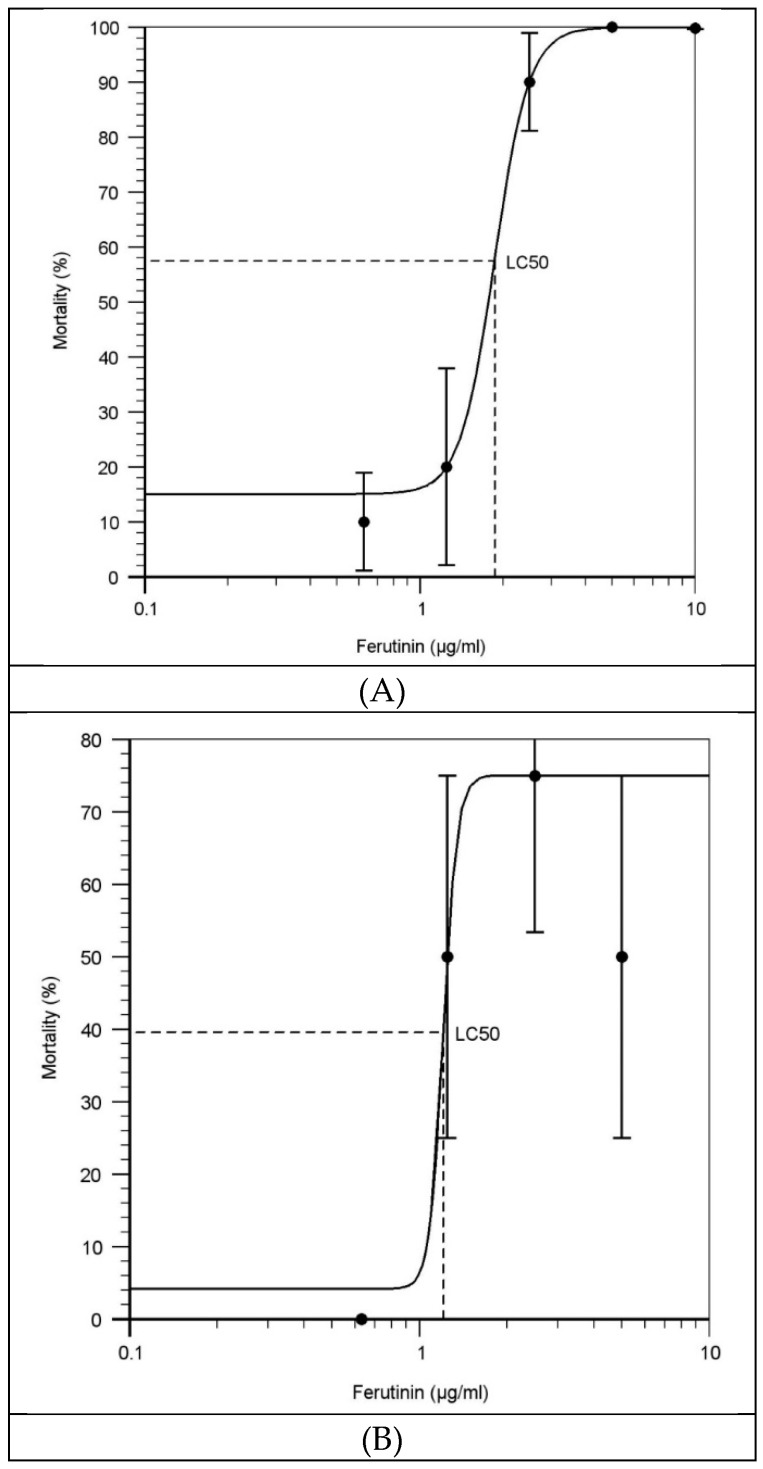

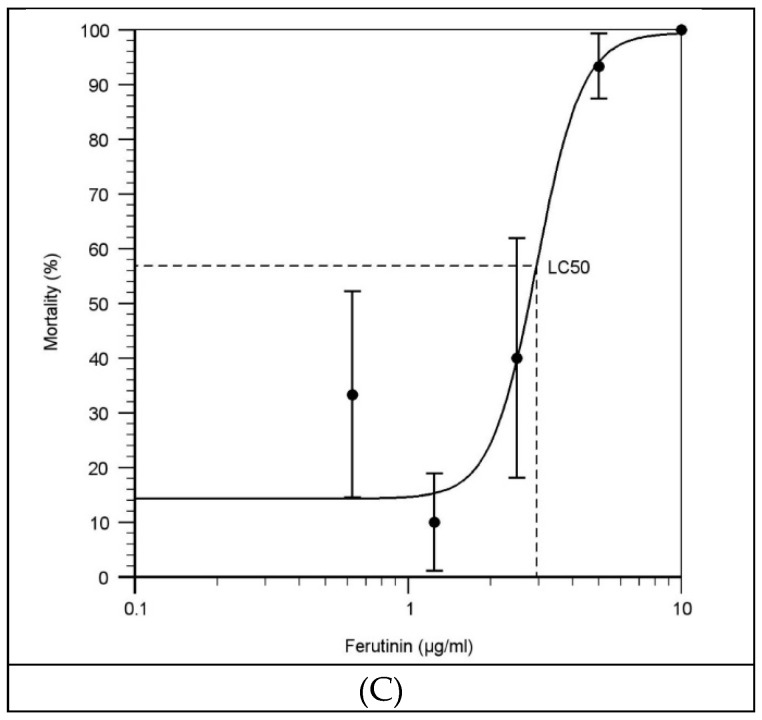
Toxicity curves to calculate the LC50 of ferutinin in 6dpf larvae which were treated at 1, 2, or 3 dpf. LC50 indicated the concentration of the ferutinin which causes mortality in 50% of the ferutinin- treated larvae at exposure time: (**A**) 1 dpf, (**B**) 2 dpf, (**C**) 3 dpf (*n* = 16 fish per group). Samples were analyzed in triplicate. Error bars are based on the standard errors of the mean (SEM). The calculations were performed using LC50 calculator [31].

The mortality percentage was calculated by counting the number of dead larvae of each clutches in each treatment at 6 dpf. However, some of the moribund fish were could tolerate treatment until 5 dpf, all of them died at 6 dpf when the survived larvae were harvested for be anesthetised and staining. According to the mortality percentage of exposed larvae, the LC50 of ferutinin at each time point (1 dpf, 2 dpf, and 3 dpf) were calculated using LC50 calculator [31]. The results of (Table 1 and Figure 4) showed that in average 50% of larvae could survive until 6 dpf while they have received approximately 1.869, 1.209 and 2.954 µg/mL of ferutinin at 1 dpf, 2 dpf, and 3 dpf, respectively. 

### 2.3. Effect of Ferutinin on Gene Expression

The expression of the genes due to RT-PCR was assigned as positive if a band appeared on gel electrophoresis. The patterns of the target genes expression were achieved by the combination of negatives and positives. For example, the pattern of the larvae which were exposed to ferutinin 1.25 μg/mL at 2 dpf represents negative, positive, positive, positive, and positive bands for *esr1*, *esrra*, *bmp2a*, *bmp2b*, and *rpl8*, respectively. The results of gene expression due to RT-PCR demonstrated that the expression pattern of the ferutinin-treated and E2-treated larvae is similar at 4 dpf when they were exposed at 2 dpf to 1.25 μg/mL of ferutinin and 10 μM of E2 (Table 2). 

**Figure 5:**
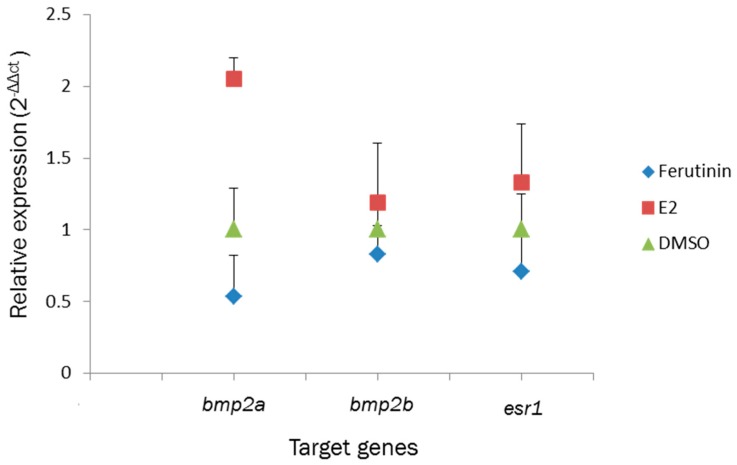
Relative quantitative expression of *bmp2a/b* and *esr1* in zebrafish larvae at 4 dpf which were treated with ferutinin (5 μg/mL) compared to 17β-estradiol (E2: 10 μM) and DMSO (0.1%) at 3 dpf. Samples were analyzed in duplicate and normalized to *rpl8* as reference gene. All values represent 2^−ΔΔ*ct*^ + SE (*n* = 30 fish) and are expressed as fold induction relative to DMSO. Abbreviation: C*t* indicates threshold cycle.

Real-time PCR was performed after RT-PCR to find effect of exposures to 5 μg/mL concentration of ferutinin, the 10 µM concentration of E2 and solvent on expression of *bmp2a*, *bmp2b*, and *esr1* compared to rpl8 as reference gene (Figure 5). The normalized results showed that the expression of *esr1* and *bmp2a/b* in larvae which were treated by the E2 was upregulated, whereas downregulation of the target genes was observed in the larvae which were treated by ferutinin 5 μg/mL. 

### 2.4. Phenotypic Readout of Treated Larval Fish

**Figure 6:**
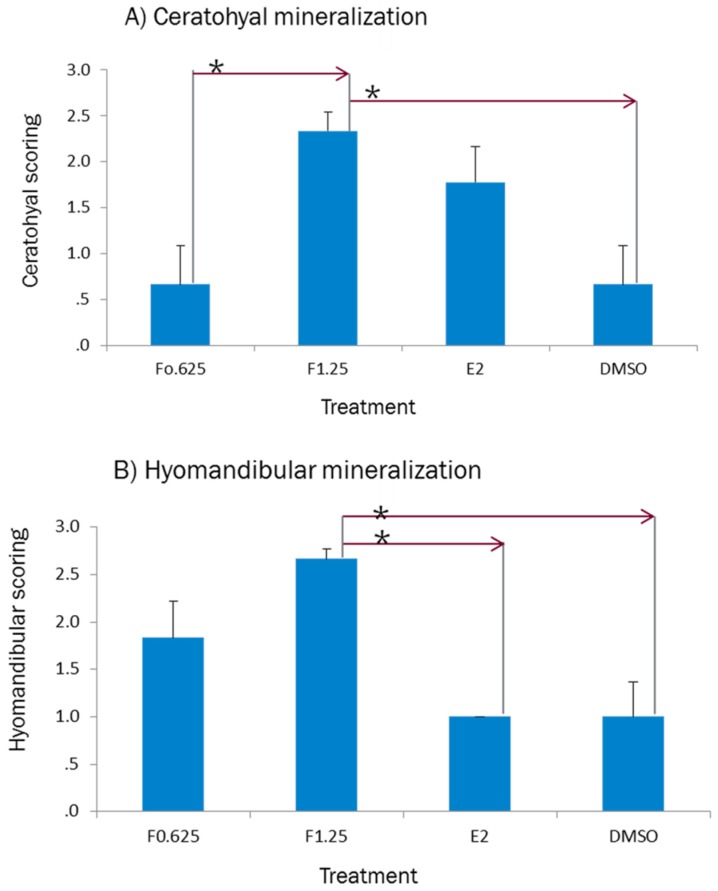
Effect of ferutinin (F: 0.625 and 1.25 µg/mL) compared to E2 (2 µM) and DMSO on bone mineralization of zebrafish larvae at 6dpf which were exposed to treatment at 2 dpf. (**A**) ceratohyal mineralization, and (**B**) hyomandibular mineralization. Abbreviations: F0.625 shows 0.625 µg/mL of ferutinin; F1.25 represents 1.25 µg/mL of ferutinin; E2 indicated 17β-estradiol. Bars represent mean +SEM (*n* = 9 fish per group). Samples were analyzed in triplicate. Dashed line and arrow indicate groups with significant difference in mean comparison (one-way ANOVA followed by Tukey’s post hoc test, * *p* < 0.05).

According to the result of the previous sections, the result of 2 dpf-treated larvae was assessed to find effect of concentration on bone mineralization (Figure 6). In general, the results of ceratohyal mineralization showed a significant difference between 1.25 μg/mL of ferutinin and DMSO as negative control. Regarding hyomandibular, the bone mineralization was increased significantly in the larvae which were treated with 1.25 μg/mL of ferutinin compared to DMSO and E2.

## 3. Discussion

### 3.1. Effect of E2 on the Phenotype of Bone Mineralization

The priority of our study was finding the effect of E2 on the phenotype of bone mineralization in zebrafish larvae because E2 was the positive control of the ferutinin section of the study and there was a lack of study in this area. However, there are several studies which traced the effect of E2 on chondrogenesis in zebrafish larvae and expression of related genes [24,25,26]. Most of them refreshed embryo media containing E2 daily until the target time of harvesting samples which was 5 dpf for analysis the changes in morphology of cartilage, but the concentration of E2 and start time of exposure, was 20 µM at 1–2 dpf, 2–3 dpf, and 3–4 dpf [24] and 0.5–5 µM at 8 hpf [25], which was 2 and 5 μM at 8 hpf [26] and 1 µM at 3 hpf [22] at different times. For example, [18] demonstrated that the high concentration of E2 caused abnormal cartilage formation and [25] showed that the major disruption occurred after treating zebrafish larvae with E2 at concentrations greater than 2 µM. We found a trend in bone mineralization of zebrafish larvae after treating with E2.

Fushimi et al. [24] showed sever curling of anteropoterior axis occurred in the larvae which were exposed to 15 µM of E2 at 1–5dpf. Moreover, they exposed larvae to 20 µM of E2 at 1–2 dpf, 2–3 dpf, and 3–4 dpf and compared the morphological changes in Meckel and ceratohyal cartilage at 5 dpf and demonstrated the changes in the larvae which were treated at 1–2 dpf was more than 3–4 dpf. Cohen et al. [25] measured the angles and length of alcian blue stained zebrafish embryos which were exposed to 0.5–5 µM of E2 at 8 hpf. In the current study, the zebrafish larvae at 3 dpf exposed to high concentration of the E2 (10 and 20 µM). The various morphological changes was observed in palatoquadrate and Meckel of the larvae which were treated with 20 µM of E2 (Figure 1H,I) and the changes in Meckel cartilage (Figure 1I) of the larvae at 6 dpf was similar to what was reported in the larvae at in 5 dpf which were exposed to 15 µM of E2 at 1–5 dpf [24] and were treated with 3 µM of E2 at 8 hpf [25]. According to the aim of the study, the changes in mineralization of the ceratohyal and hyomandibular of E2-treated larvae after exposure to 2–8 µM of E2 at 1, 2, or 3 dpf was investigated. 

Interestingly, increasing ceratohyal and decreasing hyomandibular mineralization were observed mostly at 1 dpf compared to 6hpf. Since the maternal GFP fluorescence had faded at 1 dpf and the zygotic GFP expression appeared mainly in the head region after E2 treatment but not in untreated embryos [22], the reason of observed trend could be the conversion of the source of E2 in zebrafish larvae from maternal before 1 dpf to zygotic at 1 dpf. On the other hand, the trend of ceratohyal and hyomandibular mineralization showed a positive and negative correlation to 2 μM and 8 μM in E2-treated larvae, respectively. The results of multiple comparisons showed that there is a significant difference between treating before and after 24 hpf (1 dpf) in bone mineralization of larvae which is in confirmation of the result of [23] that 24 hpf is a critical time in treating larvae with E2. 

### 3.2. Evaluation of the LC50 of Ferutinin at Each Exposure Time

The concentration of the solvent plays an important role in chemical treatment because it could produce false positive/negative results or might enhance the neurotoxicity of tested compounds dissolved in it [32,33]. DMSO 0.1% was the lowest concentration of solvent to solve target treatments which [24,25,26] applied in their zebrafish experiments. In the current study, 10 μg/mL of ferutinin was the highest concentration of the target treatment which was soluble in DMSO 0.1%. The concentration 10, 5, 2.5, 1.25, and 0.625 µg/mL of ferutinin were assumed to test the toxicity of ferutinin for the treated larvae. According to the life cycle of zebrafish, larvae have chorion at 1dpf and they are without chorion at 3 dpf. Since [23] reported chorion is not more effective on estrogen uptake and ferutinin is a phytoestrogen, in present study time of exposure to treatment was before and after 2dpf (with and without chorion). 

The LC50 was performed as the most common toxicological test to determine the relative toxicity of ferutinin to developing zebrafish larvae and was calculated for the larvae which were exposed to ferutinin at three different time points: 1 dpf, 2 dpf, and 3 dpf. 

The results indicated that most of the larvae which were treated with 5 and 10 μg/mL of ferutinin at 1 dpf and 2 dpf could not survive until 6dpf, while the well-developed larvae at 3 dpf could tolerate the higher concentration of ferutinin until 6dpf. The relative toxicity of ferutinin was calculated about 1.869, 1.209, and 2.954 µg/mL for the larvae which were treated at 1 dpf, 2 dpf, and 3 dpf, respectively and the rank order of toxicity of ferutinin for the treated larvae was 3 dpf > 1 dpf > 2 dpf. According to the developmental biology of zebrafish, the observed LC50 was expected. It seems chorion plays a protective role against ferutinin in the larvae which were treated at 1 dpf and caused lower toxicity than which were treated at 2 dpf. It could be considered as one of the difference between E2 as an estrogen and ferutinin as a phytoestrogen because [23] reported chorion is not more effective on estrogen uptake which would be an idea for future study on effect of chorion on ferutinin uptake. 

### 3.3. Effect of Ferutinin on Target Gene Expression

The larvae were exposed to treatments at three-time points: (1) 1 dpf, before hatching; (2) 2 dpf, and (3) 3 dpf, swimming. When treatment was started before 3 dpf, the yolk of the embryo was absorbed a major proportion of E2 and about prior to 48 hpf the majority of exogenous E2 was absorbed in the yolk and presumably not active in the embryonic body [23]. It means E2 was absorbed in the yolk and presumably not active in the embryonic body when the exposure time was 1 dpf; E2 is absorbed in the yolk and active in the embryonic body when the exposure time is 2 dpf and E2 is active in the embryonic body. Therefore, the exposure time and the concentration of E2 in this part of the study was 10 μM at 3 dpf to find the highest effect of positive control on the expression of related genes

In the current study, RT-PCR results in finding the effect of the start time of exposure at 1, 2, or 3 dpf on the expression of target genes at 4 dpf indicated that the pattern of appearing bands was similar in larvae which were exposed to ferutinin 1.25 μg/mL at 2 dpf and 10 µM concentration of E2. In the other words, *esrra*, *bmp2a*, *bmp2b*, and *rpl8* expressed and *esr1* did not express in treated larvae at 4 dpf. Interestingly, the results of the current study in exposing just once to the high concentration of E2 (10 µM) was similar to the low concentration of E2 in the study of [23] who showed that the expression of *esr1* was upregulated at 4dpf when the embryos treated with 1 µM of E2 at 3 hpf by refreshing every day.

On the other hand, in comparison of the effect of high concentration of ferutinin (5 µg/mL) and E2 (10 µM), ferutinin indicated the lowest relative quantitative expression of *bmp2a*, *bmp2b* and *esr1* compared to solvent and E2 in real-time PCR. According to the results, it seems ferutinin 5 μg/mL acts as an inhibitor in the expression of the target genes.

### 3.4. Effect of Ferutinin on Bone Mineralization of Zebrafish Larvae

Firstly, five concentrations of ferutinin at three time points (1 dpf, 2 dpf, and 3 dpf) were assumed to investigate the effect of ferutinin on bone mineralization of the zebrafish larvae, but just the result of two concentration of ferutinin at 2 dpf has been shown. According to the result of LC50, molecular and histology parts, it seems the result of 2 dpf-treated with ferutinin was more relevant and accurate to find effect of ferutinin on bone mineralization of tiny developing zebrafish larvae. Moreover, [23] showed a major proportion of E2 was absorbed into the yolk of the embryo when treatment was started before 3 dpf and the result of ferutinin-treated larvae at three exposure time was complicated. Interestingly, when the wild-type of zebrafish larvae were exposed to 1.25 µg/mL of ferutinin at 2 dpf bone mineralization occurred significantly and the result of histology part confirmed the result of RT-PCR. Identification the effective concentration of ferutinin on bone mineralization of zebrafish larvae at 2 dpf could be helpful for the future study to find the effect of ferutinin on healing the osteoporosis using prednisolone and dexamethasone to induce osteoporosis in transgenic zebrafish such as *tg* (*sp7:egfp*) to monitor bone formation directly. 

## 4. Materials and Methods 

### 4.1. Zebrafish Maintain, Husbandry, and Embryo Care 

All procedures involving zebrafish were performed in accordance with protocols approved by the University of Saskatchewan Committee on Animal Care and Supply and Animal Research Ethics Board (#200090108). Since the current study was the first in ferutinin-treated zebrafish studies, wild-type of zebrafish was used to optimize the method of treating for the next study on osteoporotic zebrafish. Adult wild-type zebrafish maintained in Dr. Brian F. Eames lab in an Aquatic Habitats Flow-Through System (Apopka, FL, USA) on a 14/10 day/night cycle to mimic natural conditions and were fed with alive brine shrimp and chironomids (Hikari, Hayward, CA, USA) at least once a day. The couples of wide type adult zebrafish (AB strain) mated in separated tanks, and the eggs were harvested the next day. Healthy eggs which were recognized under microscope, were transferred into 0.5× E2 (7.5 mM NaCl, 0.25 mM KCl, 0.5 mM MgSO_4_, 75 mM KH_2_PO_4_, 25 mM Na_2_HPO_4_, 0.5 mM CaCl_2_, 0.35 mM NaHCO_3_, 0.5 mg/L Methylene Blue, pH ~7.0) and were incubated at 28 °C. The dead embryos (opaque white rather than transparent) were removed and remaining embryos were rinsed once more on the day of collection and every 24 h thereafter. All embryos and larva were kept in an incubator at 28 °C when they are not being treated or cleaned.

### 4.2. Chemical Treatments

According to the purposes of current study four groups of chemicals were applied: Group 1 for investigating the effect of E2 on bone mineralization included 2, 4, 6, and 8 µM of E2; group 2 for comparing the effect of E2 and ferutinin on bone mineralization included 0.625, 1.25, 2.5, 5, and 10 µg/mL concentration of ferutinin (Sigma-Aldrich, Oakville, ON, Canada); 2 µM of E2 (Sigma-Aldrich, Oakville, ON, Canada) and 0.1% concentration of DMSO; group 3 for comparing the effect of E2 and ferutinin on target gene expression included0.625, 1.25 and 5 µg/mL concentration of Ferutinin; 10 µM of E2 and 0.1% concentration of DMSO and group 4 for real-time PCR included 5 µg/mL concentration of ferutinin; 10µM of E2 and 0.1% concentration of DMSO. 

Firstly, E2 stock solution at 10mM was prepared by dissolving 17β-estradiol in 100% DMSO. Then, the stock solution was diluted in embryo medium (EM) to the final concentration of 2 µM (540 µg/L). Furthermore, ferutinin stock solution at 10 mg/mL was prepared by dissolving ferutinin in 100% DMSO. The stock solution was added to the embryo medium (EM) to the final concentration of 0.625, 1.25, 2.5, 5 and 10 μg/mL. The final DMSO concentration after diluting treatments in embryo medium was 0.1% to avoid malformation and positive/negative false results [32,33] and negative control contained 0.1% DMSO. The treatment experiment was performed in 24-well plates, which was designed to add treatment at each time point. 

After plating chemicals, clutches of zebrafish embryos from several pairs of adult fish were divided and transferred into 24-well plate. Treating with small molecules was done with adding 3–4 larva to each well and exposed to 2 mL EM containing treatments at each time point. In the other words, they exposed to treatments based on aims of the study from 6 hpf, 1 dpf, 2 dpf, and 3 dpf.

### 4.3. Sample Selection and Screening 

Coding of each treatment was performed according to the time point of adding treatment and after raising embryos of each clutch in EM to target age, 3–4 larvae were added to each well. Then, plates including larvae of each clutched were incubated at 28 °C which is a more physiologically relevant temperature for zebrafish and increased the potency of estradiol approximately 10-fold compared to incubation at 37 °C [3].

**Figure 7:**
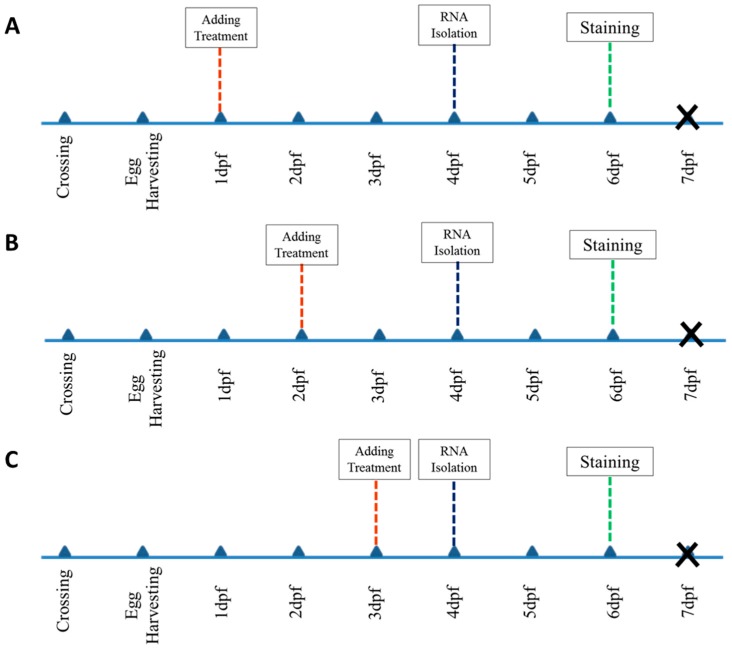
Timelines of treating larvae with small molecules, sample selection for RNA isolation and staining: (**A**) timeline of group 1 of treatment at 1 dpf, (**B**) timeline of group 2 of treatment at 2 dpf, (**C**) timeline of group 3 of treatment at 3 dpf.

The screening was performed to investigate changes in expression of the target genes and phenotype of target bones after treating larvae at each time point according to the timelines (Figure 7). For example, approximately 10 larvae of each clutch were treated at 1 dpf and remained in 24-well plate at 28 °C incubator for staining at 6 dpf. The rest of them were treated at 2 dpf and 3 dpf, respectively. Then, harvesting was performed in two steps; the 30 larvae of each treatment samples were anesthetized with 0.4% tricaine (MS-222, Sigma) and were placed into microtube at 4 dpf for RNA isolation [22] and rest of them were collected at 6 dpf for staining [18,34]. 

In order to investigate the effect of E2 on bone mineralization of zebrafish larvae, three biological replicates of approximately six larvae were collected at 6 dpf for a total of 20 larvae at each exposure time (6 hpf, 1 dpf, and 2 dpf) and treatments (0, 2, and 8 μM of E2). Moreover, an extra treatment with 8 μM of E2 at 3 dp was performed to find the effect of exposure time on bone mineralization. The ferutinin part including six treatments (0.625, 1.25, 2.5, and 5 μg/mL of ferutinin, E2, and DMSO) at three exposure time (1 dpf, 2 dpf, and 3 dpf) for analysis the mortality rates and four treatments (0.625 and 1.25 μg/mL of ferutinin, E2 and DMSO) at three exposure time (1 dpf, 2 dpf, and 3 dpf) for the molecular and histology section.

### 4.4. Calculation the LC50 of Ferutinin

The LC50 is the concentration of the compound which causes mortality in 50% of the treated-test subjects over a specific period of time [35]. The mortality of the larvae which were exposed to ferutinin (0.625, 1.25, 2.5, and 5 μg/mL) at three exposure time (1 dpf, 2 dpf, and 3 dpf) was calculated by counting the number of dead larvae of each clutches in each treatment until 6 dpf. Treated- larvae were observed daily, and dead larva were removed but were noted in final calculation. Several wells of the 24-well plates were assumed as quarantine and sick larvae or which showed malformation were transferred to quarantine with similar treatment. The linear logarithm of the exposure time and the mortality percentage until 6 dpf were plotted to reach the toxicity curves of each concentration of ferutinin. According to the mortality percentage of exposed larvae, the LC50 of the concentrations of ferutinin at each treatment group were calculated using the LC50 calculator [31]. 

### 4.5. RNA Isolation and cDNA Synthesis

Total RNA was isolated from embryos using the RNeasy mini kit (including the RNase-free DNase set) as described by the manufacturer (Qiagen, Mississauga, ON, Canada). It started with pooling around 30 larvae of each group of treatment from different clutches which were treated with ferutinin (0.625, 1.25 and 5 µg/mL) at different time points (1 dpf, 2 dpf, 3 dpf), DMSO at 1 dpf, 2 dpf, and 3 dpf, and 10 µM of E2 at 3 dpf in TRI Reagent (Sigma) and homogenized with a disposable tissue grinder pestle with matching 1.5 mL microtube (Kimble Kontes, Waltham, Massachusetts, USA). The quantity of the resulting RNA samples was assessed using a NanoDrop -Spectrophotometer (NanoDrop Technologies, Wilmington, DE, USA). cDNAs were prepared from 0.1 μg of total RNA using a RevertAid H Minus First Strand cDNA Synthesis Kit as described by manufacturer (Thermo scientific, Lithuania, EU). The 20 µL reaction volume was diluted 40-fold prior to PCR amplification. 

### 4.6. RT-PCR and Real-Time PCR

Gene- specific primers used for RT-PCR and real-time PCR which was previously reported by [26] listed in table. The candidate target genes included estrogen receptors (*esrra* and *esr1*), and potential skeletogenic targets of estrogen pathway (*bmp2a/b*) which were upregulated in the larvae which were exposed to E2 at 8 hpf with 2 and 5 μM of E2 and the media were refreshed daily until the target time (3–7 dpf) of harvesting samples [26]. Moreover, [26] validated *ppi2*, *rpl8*, and *tbp* are three suitable reference genes to accurately quantify the small differences in gene expression in developing heads of zebrafish larvae across the E2 treatment groups, but when we tested them, just *rpl8* showed constant expression and we selected it as a reference gene for real-time PCR. RT-PCR reaction conditions were performed using T100™ Thermal Cycler (Bio-Rad, Mississauga, ON, Canada). The PCR products of the larvae which were treated with ferutinin (0.625, 1.25 and 5 µg/mL) at three time points (1 dpf, 2 dpf, 3 dpf), DMSO at different time points (1 dpf, 2 dpf, 3 dpf), and E2 10 µM at 3 dpf were visualized using a 2% agarose gel electrophoresis. For RT-qPCR an ABI 7500 real-time PCR System (Applied Biosystems, ON, Canada) was employed using Power SYBR Green PCR MasterMix (Thermo Fisher Scientific, Foster City, CA, USA) following the manufacturer’s instructions. The Cycle threshold values (CT) was automatically determined by StepOne Software v2.1 (Bio-Rad, Mississauga, ON, Canada). The group of treatment including larvae which were treated with 5 µg/mL of ferutinin, 0.1% of DMSO (negative control to calculate ΔΔC*t*(ΔCt_target_ − ΔCt_DMSO_) and 2^−ΔΔ*C*t^) and 10 µM of E2 at 3 dpf were assayed in duplicate which had two replicates of cDNA of *esr1*, *bmp2 a/b*, *rpl8*, no template control (NTC) for checking the contamination in primer/probe mix or formation of primer dimer and no reverse transcriptase control (NRC) which was prepared to confirm the absence of genomic DNA contamination in RNA samples. The abundance of target and reference genes within each sample was evaluated using relative standard cure method. The data of each amplified genes were averaged and normalized to *rpl8* (reference gene to calculate ΔC*t* (Ct_Sample_ − Ct*_rpl8_*)). 

### 4.7. Staining and Scoring

Alizarin red staining was used to evaluate bone mineralized matrix deposition which is an important indicator of bone formation [18]. However, alizarin red can attach to calcium salt, staining the cartilage using alcian blue was necessary in current study to recognize and trace bone mineralization without fluorescent. Zebrafish larvae were collected and anesthetized at 6 dpf (when the cranial bone did not develop completely) to be fixed for 1hour in a 2% paraformaldehyde solution. The staining was performed based on two-color acid-free cartilage and bone staining protocol for zebrafish larvae [36]. After staining the larvae at 6 dpf, images of dorsal aspect head bone of zebrafish were taken using a DFC310 FX camera (Leica, Wetzlar, Germany) of M205 FA stereomicroscope (Leica, Wetzlar, Germany). 

**Figure 8:**
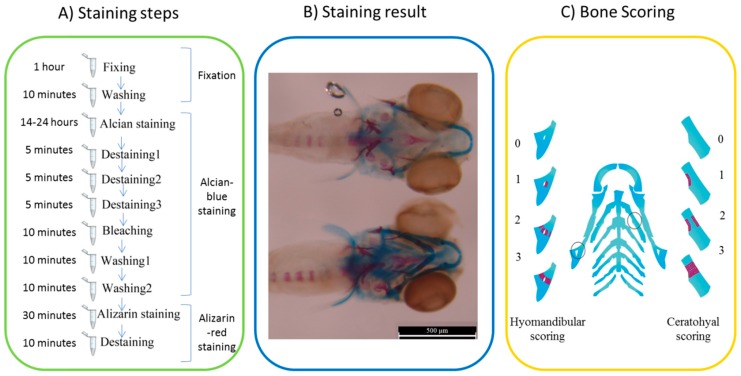
Two color staining and scoring system of the target bones in treated zebrafish larvae at 6 dpf. (**A**) Alcian-blue and alizarin-red staining steps, (**B**) sample of the stained ferutinin-treated larvae: blue parts represent ceratohyal and red parts demonstrate mineralized sections and the scale bar is 500 μM, and (**C**) ceratohyal and hyomandibular scoring system: 0 when the target area was just blue without bone mineralization, 1 for bone mineralization of one part of target cartilage, 2 for red stained two parts of cartilage but not completed, and 3 was for completely mineralized bone. Circles showed the position of the ceratohyal and hyomandibular as target bones.

Regarding cartilage and bone developments of the head skeleton in 10 dpf zebrafish [37], an atlas of zebrafish craniofacial development at a cellular resolution [38], and the schematic images of zebrafish bone development related to gene expression [39] the bone elements especially hyomandibular and ceratohyal were recognized. Pashai Ahi [39] specified the signaling pathways which were related to morphological changes of the cartilage elements, for example developmental changes in ceratohyal, palatoquadrate, and Meckel is related to estrogen pathway and the changes in hyomandibular is related to BMP pathway. The quantity of hyomandibular and ceratohyal mineralization area was determined according to the scoring system [40] of the target bones area which was stained with alizarin red staining. The applied scoring system including four scores: it was 0 when the target area was just blue without bone mineralization, 1 for bone mineralization of one part of target cartilage, 2 for red stained two parts of cartilage but not completed, and 3 was for completely mineralized bone (Figure 8). 

### 4.8. Data Analysis and Statistics

The mineralization of the target bones (hyomandibular and ceratohyal) according to scoring system was assessed for chemical concentration in special time point which was tested in triplicate (number of larvae in each well) in at least three independent experiments (number of tested clutches). The data was sorted according to the coding system using Excel. Then the ceratohyal and hyomandibular mineralization was analyzed using IBM SPSS Statistics 19 (IBM Corp., Armonk, NY, USA) as the mean ± SEM. The comparison of the means were statistically compared by one-way ANOVA (using coded treatment as independent factors and scored bones as dependent factor) for the analysis of variance and followed by a Tukey’s post-hoc test (when warranted) for multiple comparison of the means. The bone mineralization of the treated larvae were analyzed by two-way ANOVA (using the concentration and exposure time as independent factors and scored bones as dependent factor) followed by a Tukey’s post-hoc test to confirm the result of one-way ANOVA. Statistical significance for all groups of treatments was set at *p* < 0.05. The graphs were modeled by the Microsoft Excel 2010 (Microsoft Corp., Redmond, WA, USA). 

## 5. Conclusions

The results of investigating the effect of E2 on bone mineralization of zebrafish according to the start time of exposure on bone mineralization revealed a trend which indicated that there is a correlation between the concentration of E2 and ceratohyal mineralization. The PCR results in comparison effect of ferutinin in the expression of some related genes (*bmp2a/b* and *esr1*) clarified the similar effect of ferutinin1.25 μg/mL at 2 dpf and E2 10 μM at 3dpf on the pattern of the expression target genes. On the other hand, the result of real-time PCR indicated that ferutinin 5 μg/mL could inhibit the expression of target genes in treated larvae. Finally, the histology analysis to compare the effect of ferutinin and E2 on craniofacial osteogenesis demonstrated that bone mineralization occurred significantly at the 6dpf larvae which were exposed to ferutinin 1.25 μg/mL at 2 dpf. 

## Figures and Tables

**Table 1:** Effect of concentrations of ferutinin and exposure time on percentage mortality and LC50 values in treated zebrafish larvae.

Conc. of Ferutinin (µg/mL)	Exposure Time							
	1 dpf			2 dpf			3 dpf	
	Number	Mortality (%)		Number	Mortality (%)		Number	Mortality (%)
0	16	15.02		16	4.14		16	14.26
0.625	16	15.07		16	4.14		16	14.29
1.25	16	19.91		16	50		16	15.24
2.5	16	90.03		16	75		16	39.51
5	16	99.85		16	75		16	94.2
10	16	99.93		16	75		16	99.28
LC50	1.869			1.209			2.954	
Y in toxicity curves (x = conc. of ferutinin)	$15.023+\frac{99.935-15.023}{1+{\left(\frac{x}{1.869}\right)}^{-6.955}}$			$4.142+\frac{75.000-4.142}{1+{\left(\frac{x}{1.209}\right)}^{-18.105}}$			$14.259+\frac{99.431-14.259}{1+{\left(\frac{x}{2.954}\right)}^{-5.180}}$	

Exposure time: start time of exposure to treatment. dpf: day post fertilization. LC50: the concentration of the ferutinin which causes mortality in 50% of the ferutinin-treated-test larvae at exposure time. Number: total number of larvae. %: mortality percentage of larvae at each exposure time point until 6 dpf. The calculations were performed using the LC50 calculator [31].

**Table 2:** The expression pattern of target genes in 4dpf treated zebrafish larvae revealed by RT PCR.

Treatment			*Genes*		
	*Esr1*	*esrra*	*Bmp2a*	*Bmp2b*	*Rpl8*
Ferutinin 0.625 μg/mL	+	+	+	+	+
Ferutinin 1.25 μg/mL	−	+	+	+	+
Ferutinin 5 μg/mL	+	+	−	+	+
Ferutinin at 1 dpf	+	+	−	+	+
Ferutinin at 2 dpf	−	+	+	+	+
Ferutinin at 3 dpf	+	+	+	+	+
DMSO at 1 dpf	+	+	+	−	+
DMSO at 2 dpf	+	+	+	+	+
DMSO at 3 dpf	+	+	+	+	+
17β-estradiol 10µM at 3 dpf	−	+	+	+	+

+: observed band on the electrophoresis gel and gene expression. −: not appearing band on the electrophoresis gel. dpf: days post fertilization. Concentration group: ferutinin 0.625 μg/mL represents pooling of the around 30 larvae which were exposed to 0.625 μg/mL of ferutinin at 1dpf, 2 dpf or 3 dpf; Ferutinin 1.25 μg/mL indicates pooling of the around 30 larvae which were exposed to 1.25 μg/mL of ferutinin at 1dpf, 2 dpf, or 3 dpf; ferutinin 5 μg/mL demonstrates pooling of the around 30 larvae which were exposed to 5 μg/mL of ferutinin at 1 dpf, 2 dpf, or 3 dpf. Exposure time group: ferutinin at 1 dpf represents pooling of the around 30 larvae which were exposed to 0.625 and 1.25 μg/mL of ferutinin at 1 dpf; ferutinin at 2 dpf indicates pooling of the around 30 larvae which were exposed to 0.625 and 1.25 μg/mL of ferutinin at 2 dpf; ferutinin at 3 dpf demonstrates pooling of the around 30 larvae which were exposed to 0.625, 1.25, and 5 μg/mL of ferutinin at 3 dpf.

## Abbreviations

E2	17β-estradiol
hpf	Hours post fertilization
dpf	Days post fertilization
*bmp2a*	Bone morphogenetic protein 2a
*bmp2b*	Bone morphogenetic protein 2b
*esr1*	Estrogen receptor 1
*esrra*	Estrogen-related receptor 1
*rpl8*	Ribosomal protein L8
hER	Human estrogen receptor
zfERs	Zebrafish estrogen receptor
hAFSCs	Human amniotic fluid stem cells
